# Human Laminin-111-Derived AG73 Increases Proliferation, Migration, and Differentiation of Human Myoblasts: A Promising Candidate in Regenerative Medicine

**DOI:** 10.1021/acsomega.5c06289

**Published:** 2025-11-25

**Authors:** Samuel Iwao Maia Horita, Mona Bensalah, Anne Bigot, Kamel Mamchaoui, Gillian S. Butler−Browne, Daniela Gois Beghini, Wilson Savino, Capucine Trollet, Vincent Mouly, Elisa Negroni, Andrea Henriques−Pons, Ingo Riederer

**Affiliations:** 1 Fundação Oswaldo Cruz, Instituto Oswaldo Cruz, Laboratório de Inovações Em Terapias, Ensino E Bioprodutos, Rio de Janeiro 21040-360, Brazil; 2 27102 Sorbonne Université, Inserm, Institut de Myologie, Centre de Recherche En Myologie, Paris 75013, France; 3 Laboratory on Thymus Research, Oswaldo Cruz Institute, Oswaldo Cruz Foundation, Rio de Janeiro 21040-900, Brazil; 4 National Institute of Science and Technology on Neuroimmunomodulation (INCT-NIM), Oswaldo Cruz Institute, Oswaldo Cruz Foundation, Rio de Janeiro 21040-900, Brazil; 5 Rio de Janeiro Research Network on Neuroinflammation, Oswaldo Cruz Institute, Oswaldo Cruz Foundation, Rio de Janeiro 21040-900, Brazil

## Abstract

Laminin 111 (LM-111) is an extracellular matrix (ECM) glycoprotein found in basement membranes and proposed for muscle disease therapy. LM-111 treatment reduces muscle damage, restores muscle strength, alleviates inflammation, and promotes regeneration in murine and canine dystrophic models. LM-111 also improves myoblast transplantation (MT) efficacy by inducing higher proliferation, survival, dispersion, and differentiation of transplanted myoblasts. LM can undergo partial proteolysis and produce peptides called matrikines that modulate cell activity and trigger distinct biological responses from the full-length glycoproteins. In this study, we investigated the biological activity of the HuAG73 peptide, derived from the human LM, on human myoblasts, both *in vitro* and *in vivo*, using immunodeficient mice. The HuAG73 peptide offers a significant advantage over LM-111 due to its smaller size and simpler structure. HuAG73 promoted adhesion, proliferation, migration, and fusion of human myoblasts in culture. It also mimicked LM-111 in an MT assay. In conclusion, HuAG73 is a novel, relevant therapeutic candidate molecule for treating muscle diseases.

## Graphical abstract

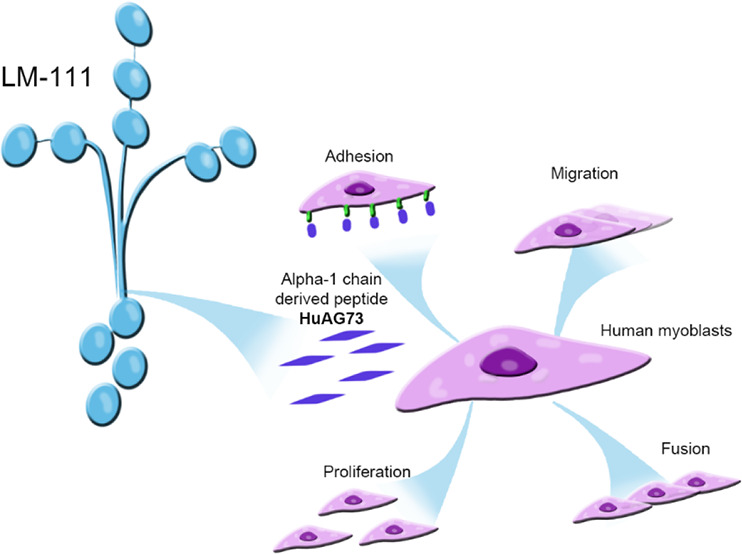

## Introduction

Laminin (LM), a major component of the basement membranes, is a heterotrimeric glycoprotein present as several distinct isoforms.^1^ The LM isoform 111 (LM-111) includes α1, β1, and γ1 chains and is widely expressed in embryonic tissues. During skeletal muscle development, both LM-111 and LM-511 (α5β1γ1) are present in the dermomyotome and myotome, being subsequently replaced upon muscle maturation by LM-211 (α2β1γ1) and LM-221 (α2β2γ1), the predominant isoforms present in the basement membrane of the adult skeletal muscle.^2,3^ LM-211 and LM-221, as other components of the extracellular matrix, transmit force and provide structural support to the cell membrane during muscle contractions by binding to the α7β1 integrin receptor and the dystroglycan complex. During muscle regeneration, LM-111 and LM-511 are re-expressed^4,5^ as well as other LM isoforms (and their corresponding receptors). In adult skeletal muscle, regeneration after trauma or disease is carried out by the muscle stem cells (satellite cell, SC). In homeostasis, these cells are maintained in a quiescent state in a niche located between the membrane of the fibers (sarcolemma) and the basal lamina, in which the presence of soluble factors and ECM components provide a microenvironment that favor stem cell maintenance.^6^ After muscle injury, the LM-111 isoform is transiently deposited within the SC niche, stimulating SC expansion and self-renewal.^5^ Both recombinant or purified LM-111 have been used as a protein-based therapeutic strategy, demonstrating an ability to increase muscle strength and decrease muscle inflammation and damage in animal models of Duchenne muscular dystrophy (DMD) and α7 integrin deficient mice.^7–11^ In addition, human myoblasts when mixed with LM-111 and injected into damaged murine muscles, showed enhanced survival, migration, and differentiation,^4,12^ suggesting a potential benefit of using LM-111 as an adjuvant for cell therapy. LMs and other ECM components can be naturally cleaved by proteinases, such as metalloproteinases (MPPs), generating peptides that can interact with cell receptors and trigger cell signaling, migration, proliferation, and adhesion.^13–15^ This digestion exposes internal peptide sequences otherwise hidden in the full-length molecule, called matricriptic sites, which can have distinct and specific effects on target cells, as revealed by the studies concerning peptides generated from the LM-111 isoform.^16^ Among the peptides derived from the mouse isoform LM-111, the 12 amino acid sequence RKRLQVQLSIRT, located at the globular domain of the α1 chain of LM-111, has been called the AG73 peptide. Since its characterization and synthesis, several biological effects have been described for this peptide, but mainly in cancer research,^16–18^ suggesting that AG73 could be a candidate for bioengineering in cell culture applications and drug delivery.^19,20^ During muscle regeneration, the expression of the LM α1 chain is followed by an increase in metalloproteinases (MMP-2 and MMP-9),^5^ which can generate the AG73 peptide from LM α1 chain. AG73, combined with hydrogel, has been shown to improve the regenerative capacity of the C2C12 mouse muscle cells after transplantation into BaCl_2_ injured mouse muscle.^21^ Other studies on the effect of LM-derived peptides on skeletal muscle regeneration are rare in the literature: the effects of AG73 on skeletal muscle regeneration have been demonstrated only using the C2C12 cell line. There are minor differences in the primary sequence between human and murine LM-111, and although the effect of the murine AG73 has been described, there are no corresponding studies using the human sequence and no comparative studies between both.^22^ During skeletal muscle repair, the LM-111 isoform is transiently produced in the SC niche^5^ and can be digested by local MMPs, releasing biologically active fragments including the AG73 peptide. Therefore, we decided to investigate the effects of this peptide on human muscle progenitor cells both *in vitro* and *in vivo* using xenotransplantation into damaged muscles of immunodeficient mice. We investigated the effects of both the murine AG73 sequence and the corresponding human sequence (HuAG73) on human myoblasts, using the intact LM-111 isoform as a control. We show here that HuAG73 stimulates the proliferation, migration, and differentiation of human myoblasts *in vitro*, as did the treatment with LM-111, whereas no such effect was observed with the murine sequence. We also observed that HuAG73 increased the proliferation, dispersion, and differentiation of human myoblasts transplanted *in vivo* into damaged muscles of immunodeficient mice, confirming a potential utilization for this LM-derived peptide to improve the regeneration of skeletal striated muscle.

## Materials and Methods

### Synthetic Peptides and LM-111 Protein

LM-111, purified from an Engelbreth-Holm-Swarm murine sarcoma basement membrane, was obtained from Sigma-Aldrich (St. Louis, USA). The murine AG73 and human AG73 synthetic peptides were obtained from GenScript (New Jersey, USA). Both peptides, murine AG73 (AG73) sequence RKRLQVQLSIRT and human AG73 (huAG73) sequence RKKLSVELSIRT, were diluted in distilled water (Gibco) (Burlington, Ontario, Canada) and stored at −80 °C. LQQRRSVLRTK was used as a scramble control peptide based on the literature.^23^

### Cell Culture

Human myoblasts were isolated from a muscle biopsy performed in a control human subject, provided by the Myobank-AFM, affiliated with the EuroBioBank, following European recommendations and French legislation (authorization AC-2019-3502). The cells were isolated as previously described^24^ and cultured in growth medium (GM) consisting of 199 medium and Dulbecco’s modified Eagle’s medium (DMEM) (Gibco) in a 1:4 ratio, supplemented with 20% FBS, fetuin (25 μg/mL), fibroblast growth factor (0.5 ng/mL), epidermal growth factor (5 ng/mL), insulin (5 μg/mL), and gentamicin (50 μg/mL). The myogenic purity of primary cells was monitored by immunocytochemistry using an anti-desmin antibody. The primary culture was immortalized by transduction of hTERT and cdk4, as already published.^25^ While both primary and immortalized human myoblasts were used for *in vitro* studies, only primary cultures were used for *in vivo* experiments. Differentiation was induced in these cultures by switching the growth medium to differentiation medium (DM), composed of DMEM supplemented with insulin (10 μg/mL) and gentamicin (50 μg/mL).

### *In Vitro* Human Myoblast Adhesion

To investigate the adhesive effects of either full-length LM-111 or peptides in human myoblasts, U-bottom 96-well plates (ref. 3799, Corning, Somerville, MA, USA) were coated overnight with either 100 μL of LM-111 (10 μg/mL) or 100 μL of peptides (murine AG73, human AG73, or scramble; 100 μg/mL). Coating concentrations were based on the literature.^26^ After the coating step, wells were blocked with 3% BSA in PBS and subsequently washed with PBS containing 0.1% BSA to minimize nonspecific cell binding. Human immortalized myoblasts were trypsinized, resuspended in serum-free DMEM (Gibco), and incubated for 20 min at 37 °C to allow recovery of surface receptors following trypsinization. Myoblasts (2 × 10^4^ cells/well) were then seeded in serum-free DMEM and incubated for 1 h at 37 °C in the previously coated U-bottom 96-well plates. For the inhibitory assay, EDTA and heparin were prepared in DMEM at twice the desired final concentration and added to the wells before cell plating, resulting in final working concentrations of 10 mM EDTA (Gibco) and 20 μg/mL heparin (Sigma). In the control wells, only DMEM (the vehicle used to dilute the inhibitors) was added. After the incubation period (1 h in the incubator), nonadherent cells were removed by washing the wells twice with PBS. Adherent cells were fixed and stained with 0.2% (w/v) crystal violet in 20% (v/v) methanol (in dH_2_O) for 10 min. Plates were rinsed with tap water, and the dye was solubilized with 10% SDS in dH_2_O. Absorbance was measured at 600 nm to quantify the number of adherent cells.

### Myoblast Proliferation Assay

Flat-bottom plates (48-well) were coated overnight with LM-111 (10 μg/mL) or with the peptides (murine AG73, HuAG73, or scramble) at 100 μg/mL. The concentration was based on previous publications.^18,27^ The cells were plated at low confluence (10^3^ cells/well) to avoid contact inhibition and differentiation and incubated at 37 °C for 1 h in GM. The medium was then replaced with 2% FBS GM and Nuclight RED (1:1000) (Sartorius—Göttingen, Germany) to reveal nuclei. The plate was maintained for 5 days in an incubator equipped with an Incucyte live imaging system (Sartorius) for image acquisition every 24 h. Images were analyzed using the Incucyte Live-Cell Analysis System (Sartorius—Göttingen).

### Myoblast Motility Assay

24-well plates were coated with LM-111 (10 μg/mL) or peptides (murine AG73, HuAG73, or scramble) at 100 μg/mL overnight. After washing with PBS, 10^4^ cells were plated at low confluence to avoid contact inhibition and differentiation and incubated at 37 °C for 2 h in GM. The medium was replaced with DMEM with 1% FBS for image acquisition with a Nikon live imaging microscope (ECLIPSE Ti2) (Nikon-Minako, Tokyo, Japan). The images were acquired every 10 min, and the cell speed analysis was performed using the TrackMate tool from Fiji ImageJ (Rasband, W.S., ImageJ NIH, Bethesda, Maryland, USA, https://imagej.net/ij/, 1997–2018; Schneider, C.A., Rasband, W.S., Eliceiri, K.W.).

### Myoblast Migration

For the migration assay, 24 mm Transwell chambers with an 8.0 μm pore polycarbonate membrane insert (Product Number 3428-Corning) were used. Human myoblasts were resuspended in 100 μL of DMEM and seeded on the top part of the insert. LM-111 (10 μg/mL) or peptides (AG73, HuAG73, or scramble; each at 100 μg/mL) were diluted in DMEM and added to the bottom chamber. The cell concentration was based on the literature.^23,27^ After 16 h, the nonmigrating cells were removed from the top chamber of the insert using a cotton swab, and the migrating cells, adhered to the bottom part of the membrane, were fixed for 10 min in PFA diluted at 4% in PBS. The cells were stained using a solution of 0.2% crystal violet as described above. The inserts were washed with tap water for visualization, and image acquisition was performed with a Primovert microscope (Zeiss-Jena, Germany). Cells were counted using the cell counter plugin of ImageJ, and the data representation was created following the Corning cell migration guidelines. LM-111 was used as a reference to normalize the migratory capacity.

### Myoblast Differentiation

12-well plates were coated overnight with LM-111 (10 μg/mL) or peptides (murine AG73, HuAG73, and scramble; each at 100 μg/mL). Then, 1.2 × 10^5^ myoblasts per well were incubated for 24 h in GM until 80% confluence was reached. To induce cell differentiation, the cells were incubated for 3 days in DM. The cells were then fixed with 100% ethanol for 10 min and incubated with 2% FBS/PBS blocking solution. To detect differentiation, a primary antibody directed against myosin heavy chain was used (MF20, Developmental Studies Hybridoma Bank, Iowa, USA, 1:20 in PBS), and binding was revealed using a secondary antibody, anti-mouse IgG Alexa-488 conjugated (A28175-Thermo). The nuclei were counterstained using a DAPI (Thermo). The fusion index was determined as the number of total nuclei located inside of the myotubes over the total number of nuclei, expressed as a percentage.

### Human Myoblast Transplantation (MT)

For cell transplantation, 2–3-month-old Rag2–/– il2rb–/– immunodeficient mice were anesthetized using ketamine (80 mg/kg) and xylazine (10 mg/kg) (Sigma), and 20 μL of Notexin (NTX) (Laxotan, France) (10 μM in NaCl 0.9%) was injected into the *tibialis anterior* (TA) muscles to induce muscle injury. At 24 h postinjury, human primary myoblasts (2 × 10^5^ cells per TA) were resuspended in 20 μL of PBS containing LM-111 (1.2 mg/mL), HuAG73 (200 μg/mL), or scramble (200 μg/mL) and injected into the TA. The concentration was based on previous publications and our *in vitro* studies.^12,23^ Twenty-one days post-cell implantation, the mice were euthanized, the muscles were collected, snap-frozen in cooled isopentane, and stored at −80 °C for further analyses. All procedures were carried out in strict accordance with the legal regulations in France and according to the European Union ethical guidelines for animal research. The protocol was approved by the Committee on the Ethics of Animal Experiments Charles Darwin N̊5 (2021091615285427 v_6). Tissue sections (5 μm) were obtained using a cryostat (Leica, Wetzlar, Germany) and stained with PBS-diluted lamin A/C (mouse IgG1 clone Jol2, Abcam, Cambridge, UK) antibody specific for human to identify human nuclei, and anti-human spectrin (clone NCL-spec1 mouse IgG2b, Novacastra-Leica) for detection of muscle fibers expressing human contractile proteins. After washing, the sections were incubated with the secondary biotinylated antibody anti-mouse IgG (Vector Laboratory, USA), followed by Streptavidin-TRITC (Becton and Dickinson, USA). Nuclei were counterstained with DAPI. The slides were mounted using a fluorescence mounting anti-fading media (Dako Agilent, Santa Clara, CA, USA), visualized using a Zeiss Apotome microscope (Germany), and the analysis was done using the ZEN software (Zeiss).

### Analysis of Muscle Sample

TA muscles were entirely cut into 5 μm sections. Sections spaced 450 μm apart along the full length of the muscle were used for quantitative analyses using lamin A/C and spectrin antibodies. The number of nuclei positive for lamin A/C was counted, and the section with the highest number of human nuclei positive for human lamin A/C (perinuclear staining) was selected for each condition. To assess regenerative capacity, the number of human spectrin (subsarcolemmal staining)-positive fibers was counted, and the maximum number of spectrin-positive fibers was determined for each TA investigated. To analyze the transversal dispersion of the injected myogenic cells, representative sections of the muscle of each group bearing the larger number of spectrin-positive fibers across the entire muscle were selected. The area of the smallest rectangle containing all the human fibers inside was calculated, and the dispersion was expressed as the surface (mm^2^) occupied by human spectrin-positive fibers in the section, using the ZEN software (Zeiss). Data were normalized to the mean value of the scramble condition, which was assigned a value of 1. Experimental conditions are shown as relative fold changes.

### Statistical Analysis

All data are reported as the mean ± standard deviation. The normalization test performed was the Shapiro–Wilk test. Comparisons between multiple groups were performed by one-way ANOVA for parametric data or by Kruskal–Wallis, and *p* < 0.05 was considered statistically significant. For comparisons between two groups, a *t*-test was used. Exceptionally, two-way ANOVA statistical analysis was used for the myoblast proliferation assay. All the analyses were done using GraphPad Prism version 8.0.

## Results

### HuAG73 and Murine AG73 Promote Myoblast Adhesion

Cell adhesion to a substrate triggers, e.g., through binding to receptors, a series of intracellular signals and can influence and control cell behavior and function, such as division, survival, and migration.^28^ To examine the effect of the LM-111-derived peptides on human myoblast adhesion, culture plates were coated with the murine AG73 and HuAG73 peptides or with LM-111, BSA, or the scramble peptide as controls. We observed that 1 h after seeding, LM-111 (Figure 1A), HuAG73 (Figure 1D), and murine AG73 (Figure 1G) can all enhance cell adhesion compared with the scrambled peptide (Figure 1J,M). To investigate which receptors were responsible for myoblast adhesion to LM-111-derived peptides, heparin (10 μg/mL) was used to inhibit heparan sulfate proteoglycan-mediated adhesion. Heparin did not affect the adhesive function of LM-111 (Figure 1B,M) compared to control (noninhibited) conditions (Figure 1A). In contrast, myoblast adhesion to HuAG73 (Figure 1E) and murine AG73 (Figure 1H) was significantly inhibited by heparin (Figure 1M). Heparin did not affect the low cell adhesion capacity of the scramble peptide (Figure 1K). EDTA was used to inhibit calcium-dependent receptors, such as integrins. Myoblast adhesion to LM-111 was significantly compromised by EDTA (Figure 1C), which partially inhibited the adhesion of these cells to HuAG73 (Figure 1F) and murine AG73 (Figure 1K). The presence of EDTA did not affect adhesion on the scramble peptide (Figure 1L,M). We also evaluated the morphology of human myoblasts in the presence of LM-111 or the peptides, using phalloidin to stain the actin cytoskeleton. As expected, coating with LM-111 promoted rapid cell spreading and cytoskeleton reorganization. Interestingly, in HuAG73- and murine AG73-coated conditions, the myoblasts showed a round shape and smaller cell size after 1 h of adhesion (Supporting Information).

**Figure 1:**
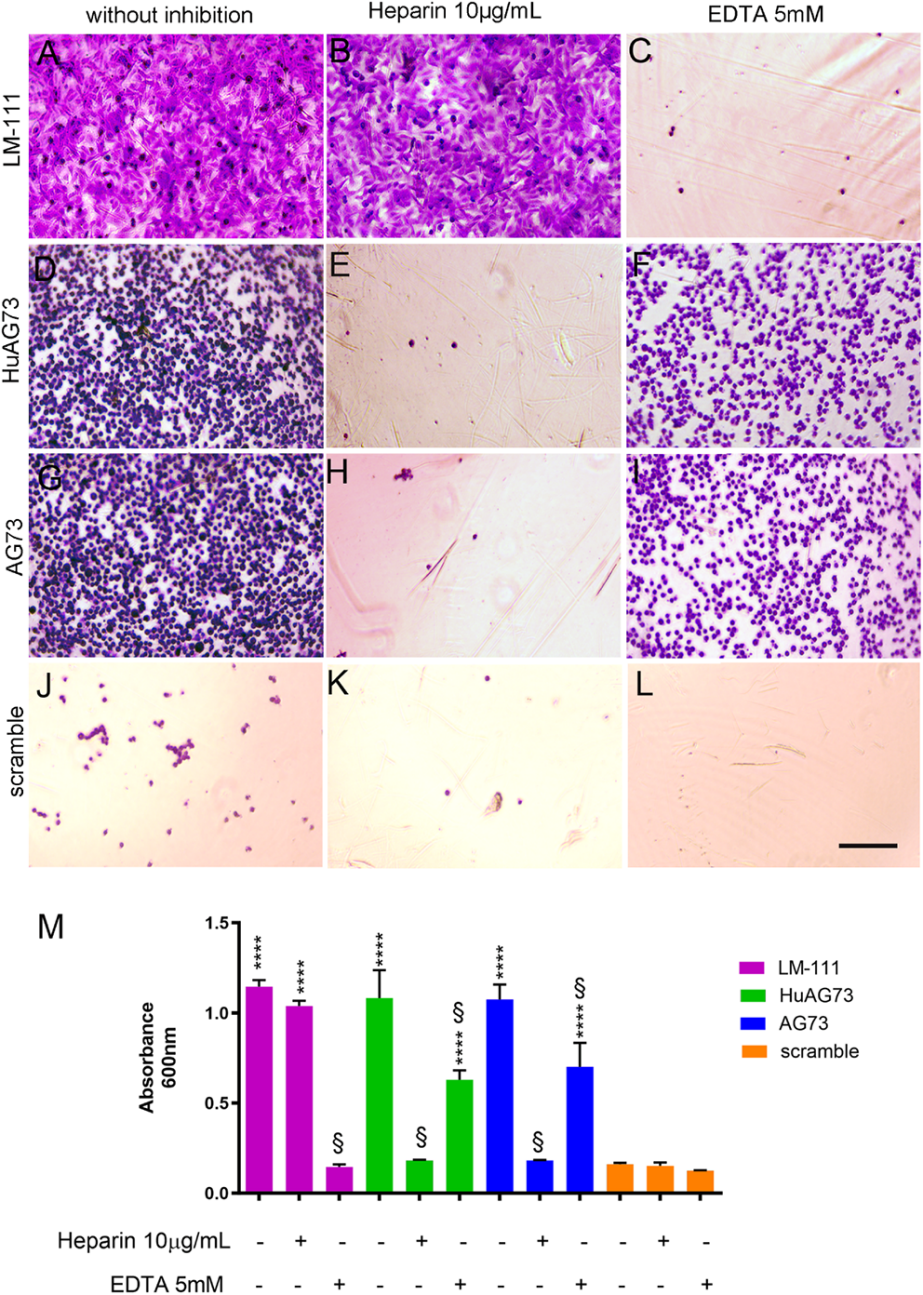
Adhesion assay on LM-111 or peptides with human myoblasts. Human myoblasts were plated on LM-111 (10 μg/mL), HuAG73, murine AG73, or scramble (100 μg/mL). The cells were stained using crystal violet, and representative images are shown from conditions (A) LM-111, (D) HuAG73, (G) AG73, and (J) Scramble. In the center, (B) LM-111, (E) HuAG73, (H) AG73, and (K) Scramble condition were treated with heparin (10 μg/mL) as an inhibitory factor for heparan sulfate receptors. On the right side, (C) LM-111, (F) HuAG73, (I) AG73, and (L) Scramble were treated with EDTA (5 mM) to evaluate the integrin-mediated adhesion. Scale bar: 50 μm. (M) Absorbance quantification at 600 nm wavelength. **** indicates *p* < 0.0001 for all experimental conditions compared to the scramble peptide control; § indicates a statistically significant difference (*p* ≤ 0.0001) between a given condition and the corresponding conditions in the absence or presence of inhibitor treatment.

### HuAG73 Increases Human Myoblast Proliferation

Binding substrates are also known to potentially modulate cell proliferation. In order to evaluate the effect of the laminin-derived peptides on human myoblast proliferation, we seeded human myoblasts onto plates precoated with LM-111 (Figure 2A), HuAG73 (Figure 2B), AG73 (Figure 2C), and scramble (Figure 2D) for 120 h. The kinetics of cell proliferation were compared to scramble for all conditions and are quantified in Figure 2E. LM-111 coating stimulated myoblast proliferation at 72, 96, and 120 h compared to the scrambled peptide, similar to previous data.^12^ The HuAG73 peptide also showed a positive effect on proliferation at 96 and 120 h. This effect was not observed with the AG73 mouse peptide. These results show that the HuAG73 peptide can increase human myoblast proliferation *in vitro* to an extent similar to that of the whole LM-111 molecule.

**Figure 2:**
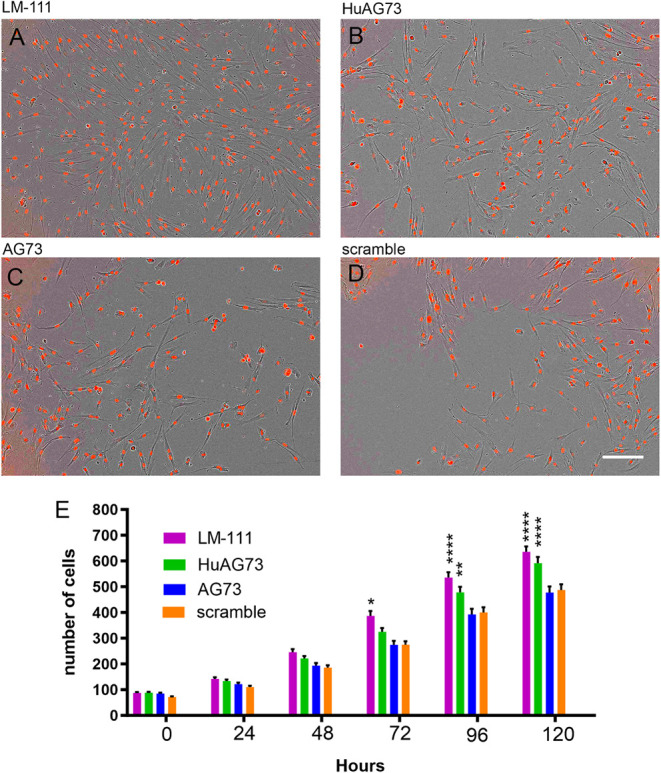
Human AG73 peptide stimulates myoblast proliferation. Representative images of 5 days of proliferation in brightfield (nuclei are stained in red). (A) LM-111, (B) HuAG73, (C) murine AG73, and (D) Scramble peptide. Scale bar: 200 μm. (E) Representative graph of the number of cells per image for time points. Statistical analysis was performed using two-way ANOVA. Each condition was compared to the scramble peptide at the corresponding time point. Statistical significance is represented as follows: *p ≤ 0.05; **p ≤ 0.01; ***p ≤ 0.001; ****p ≤ 0.0001 (*n* = 5 for each condition).

### HuAG73 Stimulates Migration and Increases Motility of Human Myoblasts

Substrates influence cell migration and motility: LM-111, HuAG73, murine AG73, and scramble peptide were added to the lower chamber of transwell plates as chemoattractants to evaluate their effect on myoblast migration. Human myoblasts were added to the upper chamber and allowed to migrate for 16 h. Confirming previous results from our group,^27,29^ LM-111 induced robust migration of human myoblasts (Figure 3A). The HuAG73 peptide also stimulated the migration (Figure 3B), whereas the murine counterpart (Figure 3C) did not stimulate the migration of myoblasts when compared to the scrambled control, although to a lesser extent than LM-111 (Figure 3A,B,E), whereas AG73 had no significant effect on migration (Figure 3C,E). To evaluate the motility of human myoblasts on the different substrates, they were seeded on culture plates coated with LM-111, AG73, HuAG73, or scramble, and live imaging was carried out for 16 h. LM-111 and HuAG73 both increased the motility (μm per minute) compared to the scramble peptide (Figure 3F). This effect was not observed with murine AG73.

**Figure 3:**
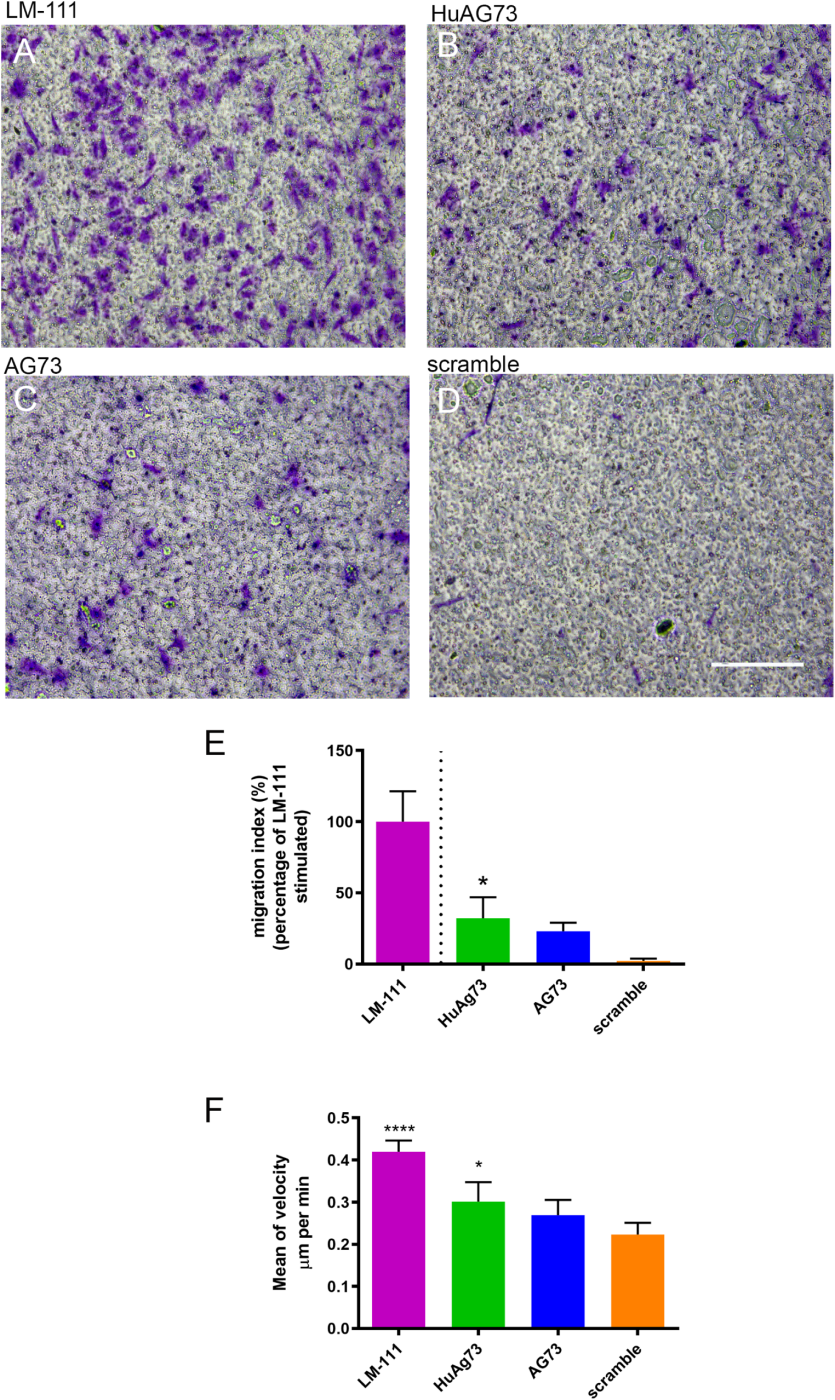
Human myoblast migration. Representative pictures of the insets of Transwell stained with crystal violet. (A) LM-111, (B) HuAG73, (C) murine AG73, and (D) scramble peptide. Scale bar: 200 μm. (E) Representative graph of migration index in the Transwell assay, where LM-111 performance was defined as the reference value (100%), and all other conditions were normalized relative to it. Statistical differences were evaluated using one-way ANOVA, with comparisons performed against LM-111. Statistical significance is indicated as follows: *p ≤ 0.05, **p ≤ 0.01, ***p ≤ 0.001, and ****p ≤ 0.0001. In (F), a representative graph of human myoblast speed (μm per min) on LM-111- or peptide-coated surfaces. Statistical analysis was performed using one-way ANOVA with all conditions compared to the scramble. Statistical significance is indicated as follows: *p ≤ 0.05, **p ≤ 0.01, ***p ≤ 0.001, ****p ≤ 0.0001 (*n* = 3 to 4 for each condition).

### LM-111 and HuAG73 Increase Myoblast Fusion

The fusion index is frequently used to evaluate the ability of myoblasts to differentiate and fuse to form multinucleated myotubes in *in vitro* experiments. We seeded human myoblasts on plates precoated with LM-111, HuAG73, murine AG73, or scramble peptide and cultured them for 3 days in differentiation medium (DM). We observed that the presence of LM-111 (Figure 4A,E) or HuAG73 (Figure 4B,E) both enhanced human muscle cell fusion *in vitro,* compared to the scramble condition. No effect was observed in myoblasts seeded on murine AG73 (Figure 4C,E).

**Figure 4:**
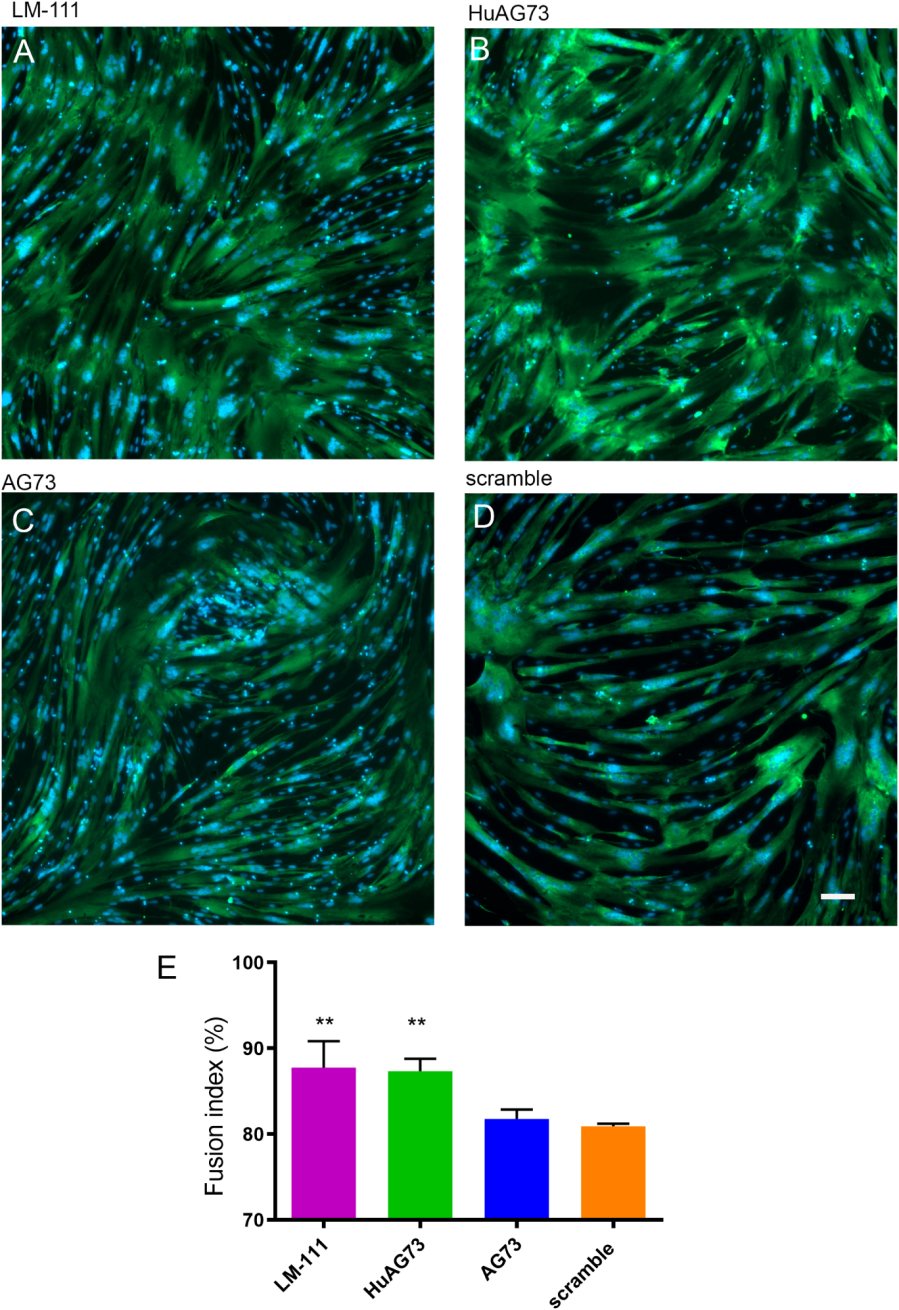
Human myoblast fusion under LM-111 or peptide stimuli. Human myoblasts were plated in 12-well plates coated with LM-111 at 10 μg/mL, murine AG73, HuAG73, and scramble peptide at 100 μg/mL. The GM was replaced with DM, and the cells were allowed to differentiate for 3 days. Cells were stained with MF20 antibody (green), and nuclei were counterstained with DAPI (blue). Representative image of (A) LM-111-, (B) HuAG73-, (C) murine AG73-, and (D) scramble peptide-treated condition; scale bar: 100 μm. (E) The fusion index (ratio of the number of nuclei in the myotubes to the total number of nuclei) was calculated and represented in the graph (E). All conditions were compared to the scramble peptide group with one-way ANOVA, where ** represents *p* ≤ 0.01 (*n* = 3 to 4 for each condition).

### LM-111 and Human AG73 Improve the Regenerative Capacity of Human Myoblasts During *In Vivo* Transplantation

The initial purpose of MT was to use muscle stem cells to participate in the host regeneration and bring a functional copy of the mutated gene, and thus of the missing protein.^30^ However, several challenges to such an approach have been reported, including extensive myoblast death, low dispersion in the host’s muscle, and early differentiation of the injected cells.^4^ Considering the positive effects of HuAG73 on human myoblasts observed in the *in vitro* experiments, we injected *in vivo* human myoblasts into Rag2–/– Il2rb–/– immunodeficient mice to assess the effect of the peptides on their *in vivo* behavior. We injected human primary myoblasts in suspension with LM-111, HuAG73, or the scrambled peptide as a negative control into the tibialis anterior (TA) muscles damaged by NTX 24 h prior to the injection. Twenty-one days after injection, the human cells and human muscle fibers were visualized and quantified using human-specific antibodies, as already published^31^ (Figure 5A). The presence of LM-111 (Figure 5B) did not increase the number of human nuclei (Figure 5E) but did increase the number of human fibers (Figure 5F) and the dispersion of the cells (Figure 5G) in the host tissue, as compared to the scrambled condition, used as a negative control (Figure 5D). These results confirm previous data showing that LM-111 can act as a coadjuvant to improve MT.^12^ Most importantly, HuAG73 (Figure 5C) increased the number of human nuclei (Figure 5E), and the number of human fibers (Figure 5F). Moreover, HuAG73 also enhanced the dispersion of the injected cells *in vivo* (Figure 5G). Overall, these experiments show that HuAG73 can enhance human myoblast proliferation, fusion, and dispersion *in vivo*.

**Figure 5:**
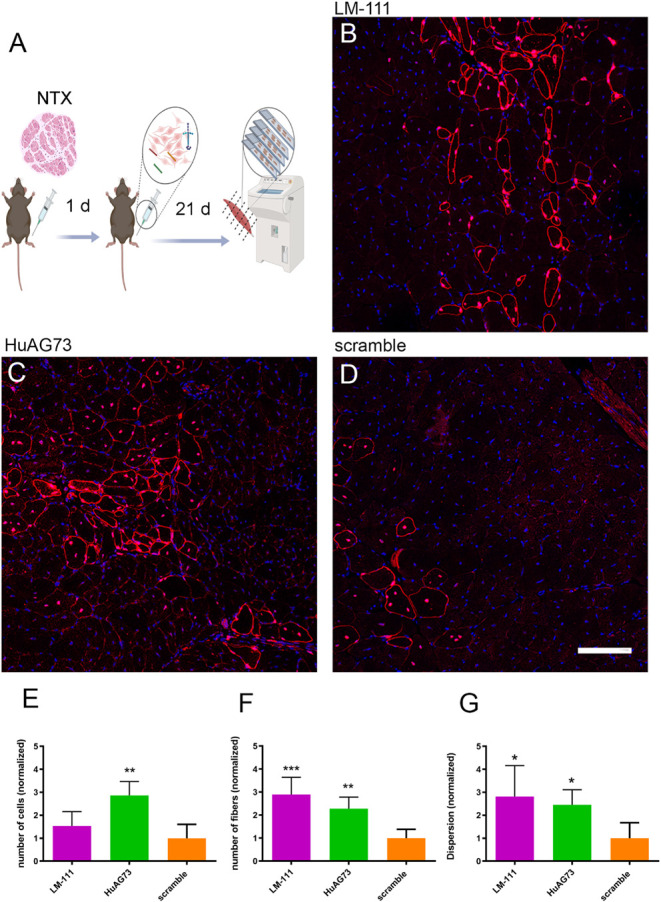
Improvement of Human MT with LM-11 and HuAG73 peptide. (A) Experimental design: human MT into immunodeficient Rag2–/– Il2rb–/– mice. Human myoblasts were treated with LM-111 (1.2 mg/mL) and HuAG73 or scramble (200 μg/mL each). The MT was performed 1 day after NTX-induced damage, and the muscles were harvested 21 days after. The human cells and fibers were detected in host muscle with human-specific anti-lamin A/C and anti-spectrin antibodies, both in red, and the nuclei in blue with DAPI. The muscle section with the largest number of fibers was chosen to perform the analysis. The figure was created with BioRender (BioRender.com). Representative images of LM-111 (B), HuAG73 (C), peptide, and Scramble peptide condition (D). Scale bar: 100 μm. (E) Graph of the analysis of human cell number counting, (F) number of human fibers, and (G) dispersion (mm^2^) of fibers in host tissue. Data were normalized to the scramble, which was set to 1. Both conditions, LM-111 and HuAG73, were compared to the scramble treatment using one-way ANOVA. Statistical significance is indicated as follows: *p ≤ 0.05, **p ≤ 0.01, ***p ≤ 0.001, ****p ≤ 0.0001 (*n* = 3 to 4 for each condition).

## Discussion

Cell therapy in skeletal muscle, using muscular progenitor cells, has been proposed as a possible treatment for muscular dystrophies and volumetric muscle loss. However, studies have shown several limitations in myoblast transplantation (MT), such as poor functional repair, extensive cell death, limited migration, and host adaptive immune response against the engrafted cells.^30^ Various strategies have been developed to stimulate the proliferation, migration, and survival of the transplanted muscle cells. After intramuscular injection, myoblasts are preferentially located in enriched laminin areas,^32^ and the treatment with purified LM-111 isoform increases survival and stimulates the proliferation and migration of human myoblasts *in vitro*.^29^ In addition, the LM-111 isoform, when used as a coadjuvant, improved the regenerative capacity of MT *in vivo* into damaged muscles.^12^ It has also been suggested that peptides can be used instead of whole proteins, since they offer the advantages of being much easier and less expensive to produce and less immunogenic. Compared with full-length proteins, peptides are more resistant to temperature and pH variations and can be more efficient in inducing specific cell signaling.^33^ Despite this, their implementation in regenerative medicine is still scarce. This deserves further investigation since transplanted stem cells treated with ECM or ECM-derived peptides have been shown to boost cellular response, and increase host tissue proliferation, migration, and differentiation.^34^ The LM-111 isoform, composed of the α1β1γ1 chains, can be cleaved enzymatically, releasing active peptides with various biological functions that have been studied initially in cancer. The AG73 peptide is a synthetic peptide derived from the amino acid sequence located at the globular c-terminus of the α1 chain, where most binding sites for cell receptors are found.^16^ Supporting our result, AG73 stimulated growth, adhesion, and neurite expansion of neural progenitor cells in culture.^35^ It is important to note that the α1 chain alone has a molecular mass of ∼337 kDa (3075 amino acids)^36^ while AG73 has only 12 amino acids, thus simplifying its clinical development. Although the use of LM-111 as a therapeutic agent to promote muscle regeneration has been investigated,^4^ few studies have addressed the effects of LM-derived peptides on myogenesis.^21^ Our work demonstrates that AG73 peptides derived from LM-111 can stimulate myogenesis in human myoblasts, similarly to the LM-111 whole molecule. In addition, our data reveal that the human AG73 (HuAG73) sequence, which had not yet been studied, has more effect on human myoblasts than its murine AG73 counterpart. Importantly, and similarly to LM-111, HuAG73 stimulates the regenerative capacity of human myoblasts when transplanted into immunodeficient mice. We also observed that both LM-111 derived peptides can promote human and mouse (data not shown) muscle cell adhesion. After 1h of incubation, the number of adhering cells was more than five times greater than the number of cells adhering to a scramble peptide. Therefore, LM-111 and AG73 peptides can induce quick and efficient adhesion, which is probably crucial in the inflammatory microenvironment created after muscle damage. Although there was no difference in the number of adherent cells between the treatment with LM-111 and the AG73 peptides, the shape of the myoblasts on LM-111, showing a flat spread phenotype, differed from the myoblasts that adhered on HuAG73 and AG73, which exhibited a round morphology. It is likely that the substrate containing AG73 and its interaction with syndecans, despite inducing strong adhesion, do not allow complete spreading, such as that provided by the isoform 111. However, our adhesion analysis was carried out after 1 h. Syndecan 4 is involved in the production of ECM molecules, including collagen, by cardiac myofibroblasts.^37^ The production of ECM that occurs after myoblasts interact with AG73 through syndecan could permit a spread morphology as observed in the LM-111 condition. An increased number of human myoblasts were observed on LM-111 and HuAG73 substrates during the *in vitro* proliferation kinetics compared to the number of cells cultured on plates coated with the murine AG73 or the scrambled peptide. Our result with the LM-111 corroborates previous findings,^12^ which showed that LM-111 enhanced human myoblast proliferation when cultivated in low sera conditions *in vitro*. Interestingly, however, in our study, the HuAG73, but not the murine AG73, promoted the proliferation of human myoblasts, which raises the question of a species-specific effect on myoblasts—whether this specificity is related to specific binding to syndecans or to other molecules. Lastly, our study showed that LM-111 and HuAG73 both increased the proliferative capacity of human myoblasts *in vivo*, a crucial step for these progenitor cells in muscle repair. Satellite cells in homeostasis represent less than 5% of the total muscle nuclei. Consequently, myoblast migration and amplification are necessary for these cells to reach damaged areas and repair and replace damaged fibers. Limited migration is considered a major hurdle for MT. Transplanted cells typically tend to stay close to the injection site,^4,38^ thus limiting their spread to the damaged area. Accordingly, stimulating migration and dispersion should improve the efficacy of the injected cells, allowing them to colonize the whole muscle.^4^ Here, we confirm data from our and other groups,^12,27,29^ identifying LM-111 as a potent inducer of myoblast migration. To analyze the effect of the peptides, we normalized the migration of human myoblasts to that on LM-111, and we observed that HuAG73 also significantly stimulated the migration. Migration is also necessary for the cell alignment that is required for proper cell fusion^39,40^ and the α6β1 integrin, a major LM receptor, is essential for myoblast differentiation. Here we show that LM-111 and HuAG73 both supported and enhanced human myoblast fusion. Finally, in order to validate our *in vitro* results in a more physiological model, we tested the effect of the HuAG73 peptide on the behavior of human cells *in viv*o. We evaluated their participation in the regeneration of the host’s muscle 21 days post-transplantation, a time point where it is possible to observe the formation of human fibers within the murine recipient tissue.^31^ We compared the effects of injecting the human primary myoblasts with HuAG73, compared to the control scramble peptide, and to LM-111, which has already been described as having stimulatory effects on the myogenic capacity of the injected cells.^12^ The higher number of human nuclei observed at 21 days after MT after HuAG73 treatment could be due to an increase in the proliferative capacity of human myoblasts since we observed that HuAG73 increased the proliferation of human myoblasts *in vitro*. However, we cannot exclude that HuAG73 also activated survival signals in the transplanted myoblasts by interacting with syndecan, hence triggering survival signaling pathways,^41^ since previous data show that a major part of the injected cells die soon after transplantation.^42^ The injection of human myoblasts with LM-111 and HuAG73 could protect the injected cells from the inflammatory milieu, stimulating survival, adhesion, and proliferation.

## Conclusion

The use of ECM-derived peptides has been described in several applications, such as in cancer research, cell biology, regenerative medicine, drug delivery, and bioengineering.^33^ Synthetic peptides used as therapeutic agents are emerging with high potential for clinical applications, and peptides can be designed with higher specificity and lower immunogenicity than whole ECM molecules, which is crucial for medical applications. Altogether, we observed that the LM-111-derived peptide HuAG73 increases the regenerative capacity of human myoblasts, similar to that of the whole molecule LM-111. This opens new clinical perspectives, including its use as an adjuvant for cell therapy in muscular dystrophies.

## Supplementary Material



## Notes

Cell morphology on LM-111 and its peptides. Fluorescence microscopy images of human myoblasts after 1 h of adhesion on wells coated with LM-111, HuAG73, and murine AG73. The cells were stained with phalloidin and DAPI (PDF)

## Abbreviations

LM-111: Laminin-111
MT: myoblast transplantation
ECM: extracellular matrix
DMD: Duchenne muscular dystrophy
MMPs: metalloproteinases
SC: satellite cell
DM: differentiation medium
