# Supplementary material for: Human Laminin-111-Derived AG73 Increases Proliferation, Migration, and Differentiation of Human Myoblasts: A Promising Candidate in Regenerative Medicine
Source: ACS Omega. 2025 Nov 25;10(48):58480–9. doi: 10.1021/acsomega.5c06289 (PMC12771415; doi:10.1021/acsomega.5c06289)
Supplement: Supplementary file 1 [file ao5c06289_si_001.pdf]

SUPLEMENTARY INFORMATION

Title

Human laminin-111-derived AG73 increases proliferation, migration, and differentiation of human myoblasts: a promising candidate in regenerative medicine

Authors

Samuel Iwao Maia Horita[1,2,3,4,5], Mona Bensalah[2], Anne Bigot[2], Kamel Mamchaoui[2], Gillian S. Butler-Browne[2], Daniela Gois Beghini[1], Wilson Savino[3,4,5], Capucine Trollet[2], Vincent Mouly[2], Elisa Negroni[2], Andrea Henriques-Pons[1] and Ingo Riederer[3,4,5]*.*

Affiliations

[1] Fundação Oswaldo Cruz, Instituto Oswaldo Cruz, Laboratório de Inovações em Terapias, Ensino e Bioprodutos, Rio de Janeiro, Brazil.

[2] Sorbonne Université, Inserm, Institut de Myologie, Centre de Recherche en Myologie, Paris, France

[3] Laboratory on Thymus Research, Oswaldo Cruz Institute, Oswaldo Cruz Foundation, Rio de Janeiro, Brazil.

[4] National Institute of Science and Technology on Neuroimmunomodulation (INCT-NIM); Oswaldo Cruz Institute, Oswaldo Cruz Foundation, Rio de Janeiro, Brazil.

[5] Rio de Janeiro Research Network on Neuroinflammation, Oswaldo Cruz Institute, Oswaldo Cruz Foundation, Rio de Janeiro, Brazil.

** corresponding authors*

Keywords: Cell therapy; Human myoblast, Xenograft, Peptides; Laminin, Biomaterials

Figure S1

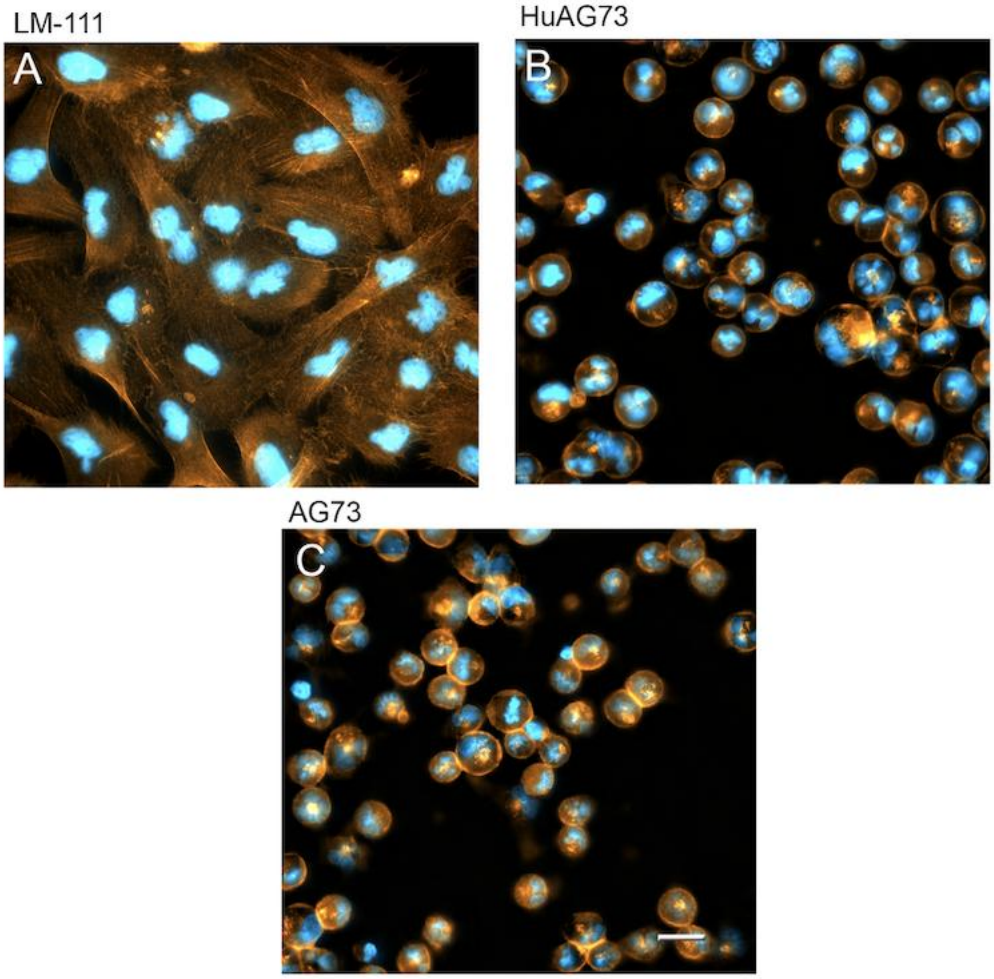

Figure S1. Cell morphology on LM-111 and its peptides. Morphology of human myoblasts after 1 hour of adhesion on wells coated with (A) LM-111, (B) HuAG73 and (C) murine AG73. Phalloidin staining (orange) was carried out to stain the actin cytoskeleton and nuclei were counterstained with dapi (blue). Scale bar: 20 µm.
